# Retraction: EBV Promotes Human CD8^+^ NKT Cell Development

**DOI:** 10.1371/annotation/1730f52d-2ba9-4f08-b330-47d71b31ae4a

**Published:** 2010-12-03

**Authors:** He Yuling, Xiao Ruijing, Ji Xiang, Li Li, Chen Lang, Xiong Jie, Xiao Wei, Wang Yujuan, Zhang Lijun, Zhou Rui, Tan Xinti, Bi Yongyi, Jiang Yan-Ping, Jin Youxin, Tan Jinquan

After an internal investigation by the Wuhan University Academic Ethics Committee, the authors of this paper requested that it be retracted. Several of the dataplots in Figure 8 contain regions that appeared to be identical, and concerns have been raised about other figures. The internal investigation could not account for the apparent duplications within Figure 8 and so the provenance and accuracy of these, and potentially other, figures cannot therefore be guaranteed. As a result, the overall integrity of this work is uncertain, and the paper was retracted from PLoS Pathogens on 03 Dec 2010. 

